# 

**Published:** 1939

**Authors:** 


### The Bristol Medico-Chirurgical Journal

A JOURNAL OF THE MEDICAL SCIENCES FOR THE WEST OF ENGLAND AND SOUTH WALES.

PUBLISHED UNDER THE AUSPICES OF

*THE BRISTOL MEDICO-CHIRURGICAL SOCIETY.*

EDITOR:

J. A. NIXON, C.M.G., M.D., F.R.C.P.

WITH WHOM ARE ASSOCIATED

E. W. HEY GROVES, D.Sc., M.S., F.R.C.S.

A. E. ILES, O.B.E., M.B., B.S., F.R.C.S.

A. RENDLE SHORT, M.D., B.S., B.Sc., F.R.C.S.

M. MELVILLE CAPPER, M.B., B.S., F.R.C.S.

E. WATSON-WILLIAMS, M.C., Ch.M., F.R.C.S., Assistant Editor

"Scire est nescire, nisi id me

Scire alius sciret."

*VOL. LVI.*

BRISTOL: J. W. ARROWSMITH LTD.
London: J. W. Arrowsmith (London) Ltd.

### Contents of Vol. LVI

*Page*

Medicine and the Drama. By LEONARD A. MOORE, M.B., CH.B. .. 1

The Management of some Obstetric Emergencies. By GILBERT I. STRACHAN, M.D., F.R.C.P., F.R.C.S., F.R.C.O.G. .. .. .. 25

Recent Advances in Surgery. By W. H. OGILVIE, M.D., F.R.C.S. 37

Some Recent Advances in Ophthalmology. By ANTHONY PALIN, B.M., B.CH., F.R.C.S. .. .. .. .. .. .. .. 85

Some Medical Considerations of Sports and Games. By J. E. LOVELOCK, B.M., B.CH. .. .. .. .. .. .. 103

Spasmodic Rhinorrhœa. By E. WATSON-WILLIAMS, M.C., CH.M. .. 109

The Seborrhœic Dermatoses. By NORMAN BURGESS, M.D., M.R.C.P. 127

Diet in Pregnancy. By J. A. NIXON, C.M.G., M.D., F.R.C.P. .. 165

The Prevention and Treatment of Prolapse. By H. J. DREW SMYTHE, M.D., M.S., F.R.C.S., F.R.C.O.G. .. .. .. .. .. 177

Basal Narcosis. By E. G. BRADBEER, M.R.C.S., L.R.C.P., D.A. .. 181

An Investigation into the Mental State of the Parents and Sibs of 1,050 Mentally Defective Persons. By RICHARD J. A. BERRY, M.D., F.R.C.S., F.R.S.E. .. .. .. .. .. .. .. 189

The Twenty-eighth Long Fox Memorial Lecture : Forest Folk : Modern Medicine in the Congo. By DR. C. C. CHESTERMAN, O.B.E., M.D., M.R.C.P. .. .. .. .. .. .. .. .. 223

Acute Appendicitis. By H. CHITTY, M.S., F.R.C.S. .. .. .. 241

War Surgery in Spain. Review by A. RENDLE SHORT, M.D., B.S., B.SC., F.R.C.S. .. .. .. .. .. .. .. .. 249

REVIEWS OF BOOKS .. .. .. .. .. .. 63, 141, 201, 252

EDITORIAL NOTES .. .. .. .. .. .. 152, 208, 258

OBITUARIES .. .. .. .. .. .. .. 70, 212, 261

MEETINGS OF SOCIETIES .. .. .. .. .. .. 156, 217

THE MEDICAL LIBRARY OF THE UNIVERSITY OF BRISTOL 72, 158, 219, 263

PUBLICATIONS RECEIVED .. .. .. .. .. 74, 162, 220, 265

LOCAL MEDICAL NOTES .. .. .. .. .. 76, 163, 222, 266

### The Medical Library of the University of Bristol

WITH WHICH IS INCORPORATED

**The Library of the Bristol Medico-Chirurgical Society.**

*The following donations have been received since the publication of the list in December,* 1938.

March, 1939.

| | |
|---|---|
| Association of American Physicians (1) .. | 1 volume. |
| Professor R. B. Brocklehurst (2) .. .. | 1 volume. |
| Brompton Hospital (3) .. .. .. | 1 volume. |
| University of Geneva (4) .. .. .. | 2 volumes. |
| Professor E. W. Hey Groves (5) .. .. | 12 volumes. |
| Colonel F. P. Mackie (6) .. .. .. | 33 volumes. |
| Dr. Paul Niehans (7) .. .. .. .. | 1 volume. |
| North Devon Athenæum (8) .. .. .. | 1 volume. |
| General Medical Council (9) .. .. .. | 2 volumes. |

Unbound periodicals have also been received from Professor E. W. Hey Groves, Professor C. Bruce Perry, Dr. G. de M. Rudolf and Dr. J. Odery Symes.

### THE ONE HUNDRED AND SIXTY-SIXTH LIST OF BOOKS.

The figures in round brackets refer to the figures after the names of the donors. The books to which no such figures have been attached have either been bought from the Library Fund or received through the *Journal.*

| | | |
|---|---|---|
| **Aiken, C. R.** | *A Concise View of all the Most Important Facts Concerning Cow-Pox* 2nd Ed. (8) | 1801 |
| **Berkeley, Sir Comyns** | *A Handbook of Midwifery* .. 10th Ed. | 1938 |
| **Berry, R. J. A.** | *A Cerebral Atlas* | 1938 |
| **Böhler, L.** | *Technik der Knockenbruchbehandlung.* 2 Vols. (5) | 1937 |
| **Campbell, A., and Poulton, E. P.** | *Oxygen and Carbon Dioxide Therapy* 2nd Ed. | 1938 |
| **Claye, A. M.** | *The Evolution of Obstetric Analgesia* | 1939 |
| **Davis, A. A.** | *Dysmenorrhoea* | 1938 |
| **De Kruif, P.** | *Microbe Hunters* | 1938 |
| **East, T.** | *Cardiovascular Disease in General Practice* | 1938 |
| **Elmer, A. W.** | *Iodine Metabolism and Thyroid Function* | 1938 |
| **Fulton, J. F.** | *Physiology of the Nervous System* | 1938 |
| **Gleichgewicht, A.** | *Les hernies de l'estomac à travers l'hiatus œsophagien* (4) | 1937 |
| **Granville, A. B.** | *The Spas of England and Principal Sea-Bathing Places—Midland Spas* | 1841 |
| **Ham, I. Burnett** | *Handbook of Sanitary Law* .. 12th Ed. | 1938 |
| **Hoffheinz, S.** | *Die Luft- und Fettenembolie* (5) | 1933 |
| **Hoyle, C., and Vaizey, M.** | *Chronic Miliary Tuberculosis* | 1937 |
| **Kappis, M.** | *Vorbeugung und Bekämpfung der Operationsgefahren* (5) | 1933 |
| **Kayne, G. G.** | *The Control of Tuberculosis in England* | 1938 |
| **Ker, C. B.** | *Manual of Fevers.* Revised by F. L. Ker 4th Ed. | 1939 |
| **Kienbock, R.** | *Röntgendiagnostik der Knochen- und Gelenkkrankheiten.* Hefte 4–5 (5) | 1934–6 |
| **Lanza, A. J.** | *Silicosis and Asbestosis* | 1938 |
| **Lomholt, S.** | *Die Moderne Finsenbehandlung* (5) | 1934 |
| **Loup, C.** | *Contribution à l'étude toxicologique de trente-trois inocybe* (4) | 1938 |
| **Moberly, J.** | *The Health of the Nation and Deficiency Diseases* | 1938 |
| **Moon, V. H.** | *Shock and Related Capillary Phenomena* | 1938 |
| **Niehans, P.** | *Die endokrinen Drusen des Gehirns* (7) | 1938 |
| **Oberholzer, J.** | *Röntgendiagnostik der Gelenke mittels Doppelkontrastmethode* (5) | 1938 |
| **Pye, W.** | *Surgical Handicraft.* Ed. by Hamilton Bailey 11th Ed. | 1939 |
| **Rogers, Sir Leonard** | *The Truth about Vivisection* (2) | 1937 |
| **Rogerson, C. H.** | *Play Therapy in Childhood* | 1939 |
| **Rowlette, R. J.** | *The Medical Press and Circular,* 1839–1939 | 1939 |
| **Vianna, B.** | *Conférences d'orthopédie et de chirurgie* (5) | 1936 |
| **Weisz, E.** | *Diagnostik mit freiem Auge* .. 4th Ed. (5) | 1933 |

| | | | |
|---|---|---|---|
| **Whitla, Sir W.** .. | *Dictionary of Treatment* .. .. | 8th Ed. | 1938 |
| **Wile, I. S.** .. .. | *Personality Development and Social Control in Terms of Constitution and Culture* .. .. | | 1939 |
| **Wilson, P. D. (ed.)** .. | *Experience in the Management of Fractures and Dislocations* .. .. .. .. .. .. | (5) | 1938 |
| **Zeno, L. O.** .. .. | *Fractura del cuello del fémur* .. .. | (5) | 1934 |

TRANSACTIONS, REPORTS, JOURNALS, ETC.

| | | |
|---|---|---|
| Brompton Hospital Reports. Vol. VII. .. .. .. .. .. .. | (3) | 1938 |
| Dental Register, 1939 .. .. .. .. .. .. .. .. .. .. .. .. | (9) | 1939 |
| Index Catalogue of the Library of the Surgeon General's Office. 4th Series, Vol. III .. .. .. .. .. .. .. .. .. .. .. .. .. .. | | 1938 |
| Medical Register, 1939 .. .. .. .. .. .. .. .. .. .. .. .. | (9) | 1939 |
| St. Thomas's Hospital Reports. Vol. III, 2nd Series .. .. .. .. | | 1938 |
| Transactions of the Association of American Physicians. Vol. LIII | (1) | 1938 |
| Transactions of the Ophthalmological Society of the United Kingdom. Vol. LVIII, Part II .. .. .. .. .. .. .. .. .. .. .. .. .. | | 1938 |
| Tropical Diseases Bulletin, Vols. 1–33 .. .. .. .. .. .. | (6) | 1912–1936 |

### Publications Received

From MESSRS. BAILLIÈRE, TINDALL & COX :

*Aids to Forensic Medicine and Toxicology.* By W. G. AITCHISON ROBERTSON, M.D., D.Sc., F.R.C.P.E. 11th Edition. Price 3s. 6d. net.

*Modern Treatment in General Practice, Yearbook,* 1939. Price 12s. 6d.

*British Medical Societies.* Edited by Sir D'ARCY POWER, K.B.E., F.R.C.S. Price 10s. 6d.

*The Surgery of Pain.* By RENÉ LERICHE, M.D., LL.D., F.R.C.S. Translated and edited by ARCHIBALD YOUNG, B.Sc., M.B., C.M., F.R.F.P.S.G., F.A.C.S., M.D. Price 21s.

From JOHN BALE MEDICAL PUBLICATIONS LTD. :

*Rheumatism.* By H. WARREN CROWE, D.M., B.Ch., M.R.C.S., L.R.C.P.

From THE INTERNATIONAL TIN RESEARCH AND DEVELOPMENT COUNCIL :

Publication No. 89, *Tin and its Alloys in Dental Practice.* By F. C. THOMPSON, D.Met., M.Sc., and N. WILD, L.D.S.

From MESSRS. H. K. LEWIS & CO. LTD. :

*Infra-Red Irradiation.* By WILLIAM BEAUMONT, M.R.C.S., L.R.C.P. 2nd Edition. Price 6s. 6d.

*Recollections of Student Life and Later Days.* By C. J. BOND, C.M.G., F.R.C.S., F.L.S. Price 1s.

*Treatment by Manipulation.* By A. G. TIMBRELL FISHER, M.C., M.B., Ch.B., F.R.C.S., being the 3rd Edition of *Manipulative Surgery.* Price 12s. 6d.

*Tests for the Hearing of Speech by Deaf People.* By D. B. FRY, B.A., and P. M. T. KERRIDGE, Ph.D., M.R.C.P. Price 4s. 6d.

*Gould's Pocket Pronouncing Medical Dictionary.* By the late GEORGE M. GOULD, A.M., M.D. 11th Edition. Revised by C. V. BROWNLOW. Price 10s. 6d.

*Fractures and Dislocations in General Practice.* By JOHN HOSFORD, M.S., F.R.C.S. Price 12s. 6d.

*Uterus Masculinus.* By J. A. LEO MAGEE, M.B. Price 5s.

*Sanitary Law in Question and Answer.* By CHARLES PORTER, M.D., B.Sc., M.R.C.P., and JAMES FENTON, C.B.E., M.D., M.R.C.P., D.P.H. 4th Edition. Price 10s.

*Treatment in General Practice.* Vol. III. *Anæsthesia: Surgery.* (Articles republished from *The British Medical Journal*). Price 10s. 6d.

From THE MEDICAL PRESS & CIRCULAR:

*The Medical Press and Circular, 1839–1939.* By ROBERT J. ROWLETTE, M.D., F.R.C.P.I.

From OXFORD UNIVERSITY PRESS (Humphrey Milford):

*The Physiology of Anesthesia.* By HENRY K. BEECHER, A.B., A.M., M.D. Price 17s. 6d. net.

*Clinical Electrosurgery.* By GUSTAVUS M. BLECH, G.C.S., G.C.G., M.D., LL.D. Price 21s.

*The Evolution of Obstetric Analgesia.* By ANDREW M. CLAYE, M.D., F.R.C.S., F.C.O.G. Price 6s. net.

*The Postnatal Development of the Human Cerebral Cortex.* Vol. I. By J. LEROY CONEL.

*Handbook of the Vaccine Treatment of Chronic Rheumatic Diseases.* By H. WARREN CROWE, D.M., B.Ch., M.R.C.S., L.R.C.P. 3rd Edition. Price 3s. 6d.

*Carbon Monoxide Asphyxia.* By CECIL K. DRINKER, M.D., D.Sc. Price 25s.

*The Evolution and Organization of the University Clinic.* By SIMON FLEXNER, M.D. Price 3s. 6d.

*Orthopœdic Appliances.* By HENRY H. JORDAN, M.D. Price 21s.

*Ker's Manual of Fevers.* Revised by FRANK L. KER, B.A., M.B., Ch.B. 4th Edition. Price 12s. 6d. net.

*Silicosis and Asbestosis.* By various authors. Edited by A. J. LANZA, M.D. Price 25s. net.

*Shock and Related Capillary Phenomena.* By VIRGIL H. MOON, A.B., M.Sc., M.D. Price 21s. net.

*Cranio-Cerebral Injuries.* By DONALD MUNRO, A.B., M.D., F.A.C.S. Price 21s. net.

*Play Therapy in Childhood.* By C. H. ROGERSON, M.D., M.R.C.P., D.P.M. Price 3s. 6d. net.

*The March of Medicine.* By RAY LYMAN WILBUR, M.D. Price 12s. 6d.

*Personality Development and Social Control in Terms of Constitution and Culture.* By IRA S. WILE, M.S., M.D. Price 3s. 6d. net.

From MESSRS. JOHN WRIGHT & SONS LTD.:

*British Journal of Surgery.* January, 1939. 12s. 6d. net. (Annual Subscription 42s.).

*Asthma.* By FRANK COKE, F.R.C.S. 2nd Edition. Price 15s.

*Pye's Surgical Handicraft.* Edited by HAMILTON BAILEY, F.R.C.S. 11th Edition. Price 21s. net.

*Synopsis of Medicine.* By H. LETHEBY TIDY, M.A., M.D., B.Ch., F.R.C.P. 7th Edition. Price 21s. net.

### Local Medical Notes

The twenty-eighth Long Fox Memorial Lecture will be delivered at the University on Tuesday, November 14th, 1939, by Dr. Clement C. Chesterman. Subject "Forest Folk: Modern Medicine in the Congo."

EXAMINATION RESULTS

**Conjoint Board.**—*Final Examination, Pass:* *Joan E. Mackworth (Midwifery), A. A. Stonehill, F. R. Hurford, M. Dworkis, Sara M. Field-Richards, Betty Fox, Mary R. More (Pathology), Barbara Cunningham, *R. T. Moore (Surgery).

*First Examination. Pass:* Margot H. Dickson (Physiology), B. C. E. Aldous (Pharmacology and Materia Medica).

**University of Cambridge.**—M.B., B.S. (*Final Examination*). *Pass:* K. G. Bergin.

* Qualified.

**Colston Medical Research Fellowships.**—The Council of the Colston Research Society, Bristol, offers one or more Fellowships for 1939–40 of not less than £100 per annum for Research work primarily in Clinical subjects, though other medical subjects are not excluded. The work must be carried out in the University of Bristol or in any of the Clinical Institutions associated with the University.

Awards will be made in June or July, 1939. Applications should reach the Hon. Secretary of the Colston Research Society, 12 Small Street, Bristol 1, not later than June 1st.

### Bristol Medico-Chirurgical Society.

### President.

L. A. MOORE, M.B., Ch.B. Brist.

### President-Elect.

C. CORFIELD, M.D. Durh.

### Committee.

*Ex-Officio* { J. A. NIXON, *C.M.G.*, B.A., M.D. Cantab., F.R.C.P. (Editor of *Journal*).

(Vacant), (Hon. Librarian).

*Elected.*

C. A. MOORE, M.B., M.S. Lond., (Ex-President).
R. C. CLARKE, *O.B.E.*, M.B., Ch.B.Brist.
H. H. CARLETON, M.A., M.D., B.Ch.Oxon.
C. CORFIELD, M.D. Durh.
A. L. TAYLOR, M.D. Lond.
P. S. MARTIN, *M.C.*
T. MILLING, M.B., Ch.B. Belf.
W. V. WOOD, *M.C.*, B.A. Cantab.
H. L. SHEPHERD, M.B., Ch.M. Brist.
W. A. JACKMAN, F.R.C.S.

### Honorary Secretary.

R. V. COOKE, Ch.M.

### Honorary Treasurer.

A. E. ILES, *O.B.E.*, M.B., B.S. Lond., F.R.C.S.

### University Library Sub-Committee.

A. R. SHORT, B.Sc., M.D. Lond., F.R.C.S.
C. BRUCE PERRY, M.D. Brist., F.R.C.P.
J. H. DREW SMYTHE, *M.C.*, M.S., M.D. Lond., F.R.C.S., F.C.O.G.

Medical Practitioners wishing to join the Society are requested to communicate with the Hon. Secretary, Mr. R. V. COOKE, Failand Lodge, Guthrie Road, Clifton. The Annual Subscription is £1 1s., payable to Hon. Treasurer.

---

*Extracts from Journal By-laws.*

4.—The *Journal* shall be delivered free to Subscribers and also to Members of the Society whose subscriptions are not in arrear.

6.—The Annual Subscription to the *Journal* is 10/6, payable to Hon. Treasurer.

---

Communications intended for insertion in the *Journal* should be sent to the Editor, Dr. J. A. NIXON, 7 Lansdown Place, Clifton, Bristol.

Books for Review and Exchange Journals should be sent to the Assistant-Editor, *Bristol Medico-Chirurgical Journal*, Medical Library, The University, Bristol. Reviews should be returned with the books to the Assistant Editor at 65 Pembroke Road, Clifton, Bristol 8.

Communications referring to the delivery of the *Journal* should be sent to the Editorial Secretary, A. L. Flemming, 48 Pembroke Road, Clifton, Bristol.

Cases for Binding Vols. I—LV are now ready, and may be obtained price 1/9 each (postage extra), upon application to J. W. ARROWSMITH LTD., Quay Street, Bristol; or the *Journal* will be bound, including Case, for 7/– per volume. The inclusive index for Volumes I—L may be obtained, price 3/– (postage extra).

### Bristol Medico-Chirurgical Society.

### PAST PRESIDENTS.

1874—75. FREDERICK BRITTAN, M.D., B.S. Dub.
1875—76. ROBERT WILLIAM COE.
1876—77. SAMUEL MARTYN, M.D. Ed.
1877—78. AUGUSTIN PRICHARD, M.D. Berlin.
1878—79. HENRY EDWARD FRIPP, M.D. Würzburg.
1879—80. WILLIAM MICHELL CLARKE.
1880—81. JOSEPH GRIFFITHS SWAYNE, M.D. Lond.
1881—82. EDWARD LONG FOX, M.D. Oxon.
1882—83. JAMES GEORGE DAVEY, M.D. St. And.
1883—84. WILLIAM JOHNSTONE FYFFE, M.D., B.A. Dub.
1884—85. GEORGE FORSTER BURDER, M.D. Aberd.
1885—86. WILLIAM HENRY SPENCER, M.D., M.A. Cantab.
1886—87. FRANCIS POOLE LANSDOWN.
1887—88. LEMUEL MATTHEWS GRIFFITHS.
1888—89. ROBERT SHINGLETON SMITH, M.D., B.Sc. Lond.
1889—90. NELSON CONGREVE DOBSON, Ch.M. Brist.
1890—91. SAMUEL HENRY SWAYNE.
1891—92. FRANCIS RICHARDSON CROSS, M.B. Lond., LL.D. Brist.
1892—93. EDWARD MARKHAM SKERRITT, M.D., B.S., B.A. Lond.
1893—94. JAMES GREIG SMITH, M.B., C.M., M.A. Aberd., F.R.S.E.
1894—95. ALFRED JAMES HARRISON, M.B. Lond.
1895—96. ARTHUR WILLIAM PRICHARD.
1896—97. ALFRED EDWARD AUST LAWRENCE, M.D., C.M. Aberd.
1897—98. JOHN EDWARD SHAW, M.B., C.M. Ed.
1898—99. ROBERT ROXBURGH, M.B. Ed.
1899—00. WILLIAM HENRY HARSANT.
1900—01. DAVID SAMUEL DAVIES, M.D. Lond.
1901—02. BARCLAY JOSIAH BARON, M.B., C.M. Ed.
1902—03. GEORGE MUNRO SMITH, M.D. Brist.
1903—04. JAMES PAUL BUSH, C.M.G., C.B.E., Ch.M. Brist
1904—05. REGINALD EAGER, M.D. Lond.
1905—06. JOHN DACRE.
1906—07. JAMES TAYLOR.
1907—08. HENRY WALDO, M.D., C.M. Aberd.
1908—09. JOHN MICHELL CLARKE, M.D., M.A. Cantab.
1909—10. JAMES SWAIN, C.B., C.B.E., M.D., M.S. Lond.
1910—11. BERTRAM MITFORD HERON ROGERS, M.D., B.Ch., B.A. Oxon
1911—12. CHARLES ALEXANDER MORTON.
1912—13. WALTER CARLESS SWAYNE, M.D., B.S. Lond. and Brist.
1913—14. PATRICK WATSON-WILLIAMS, M.D. Lond., M.D. (Hon. Causa) Brist.
1914—15. WILLIAM HARRY CHRISTOPHER NEWNHAM, M.A., M.B. Cantab.
1915—16. GEORGE PARKER, M.A., M.D. Cantab., LL.D. Brist.
1916—17. FRANCIS HENRY EDGEWORTH, M.A., M.D. Cantab., D.Sc. Lond.
1917—18. FREDERICK LACE.
1918—19. ROBERT GUTHRIE POOLE LANSDOWN, M.D., B.S. Durh.
1919—20. I. WALKER HALL, M.D., Ch.B. Vict.
1920—21. LEOPOLD ERNEST VALENTINE EVERY-CLAYTON, M.D. Lond.
1921—22. CYRIL HUTCHINSON WALKER, B.A., M.B. Cantab.
1922—23. JOHN ALEXANDER NIXON, C.M.G., B.A., M.D. Cantab.
1923—24. JOHN LACY FIRTH, M.D., M.S. Lond.
1924—25. JOHN ODERY SYMES, M.D. Lond.
1925—26. THOMAS CARWARDINE, M.S. Lond.
1926—27. ARTHUR LAUNCELOT FLEMMING, M.B., Ch.B. Brist.
1927—28. WALTER KENNETH WILLS, M.A., M.B., B.Ch. Cantab.
1928—29. HENRY LAWRENCE ORMEROD, M.D., B.Ch. R.U.I.
1929—30. CHARLES FERRIER WALTERS.
1930—31. DAVID CHARLES RAYNER, Ch.M. Brist.
1931—32. JOHN ROGER CHARLES, M.A., M.D. Cantab.
1932—33. ERNEST WILLIAM HEY GROVES, M.D., M.S., D.Sc., B.Sc. Lond.
1933—34. HERBERT ELWIN HARRIS, B.A., M.B. Cantab.
1934—35. A. RENDLE SHORT, B.Sc., M.D. Lond.
1935—36. ARTHUR ERNEST ILES, O.B.E., M.B., B.S. Lond.
1936—37. RICHARD CHRISTOPHER CLARKE, O.B.E, M.B. Lond.
1937—38. CLIFFORD A. MOORE, M.B., M.S. Lond.

1939.

### LIST OF SUBSCRIBERS

TO THE

### Bristol Medico-Chirurgical Journal.

### HONORARY MEMBER OF THE SOCIETY.

SWAIN, JAMES, *C.B.*, *C.B.E.*, M.D., M.S. Lond. .. .. .. Wyndham House, Easton-in-Gordano.

### SUBSCRIBERS.

*Those marked * are Members of the Society.*

| | |
|---|---|
| *ABBOTT, Miss E. K., M.B., Ch.B. (Otago) .. | X-Ray Department, Bristol General Hospital. |
| *ADAMS, A. W., M.S. Lond. .. .. .. .. | Rodney House, Clifton, Bristol 8. |
| ADAMS, J. B. .. .. .. .. .. .. .. .. | Kent Lodge, Eastbourne. |
| *ADAMS, S.B., Ph.D., M.B., Ch.B. Brist. .. .. | 19 Charlotte Street, Bristol 1. |
| *ALDWINCKLE, HELEN M., M.B., Ch.B. Brist. .. | 1 Manor Road, Fishponds, Bristol. |
| *ALEXANDER, A. J. P., M.D. Belf. .. .. .. | Winsley Sanatorium, near Bath. |
| *ALEXANDER, D. A., M.B., Ch.B. Vict. .. .. | 112 Pembroke Road, Clifton, Bristol 8. |
| ALEXANDER, G. L., B.A., M.B., B.Ch. Cantab. | West African Medical Service, Laura, via Accra, Gold Coast. |
| *ALFORD, A. F., M.B., Ch.B. Brist. .. .. .. | Board of Education, Whitehall, London, S.W. 1. |
| ALFORD, H. J., M.D. Lond. .. .. .. .. | 4 Park Terrace, Taunton. |
| *ARMSTRONG, JEAN, M.B., Ch.B. Brist. .. .. | Succoth, Duckmoor Road, Ashton Gate, Bristol 3. |
| ASHTON, L. P., M.B., Ch.B. Brist. .. .. .. | Kapsowar, P.O. Eldoret, Kenya, East Africa. |
| *AUBREY, T., M.B. Lond. .. .. .. .. .. | Bitton, near Bristol. |
| *BAIN, W. .. .. .. .. .. .. .. .. .. | 1 Greville Road, Southville, Bristol 3. |
| *BAKER, LILY A., M.D., B.Ch., B.A.O., R.U.I. | 23 Pembroke Road, Clifton, Bristol 8. |
| *BARBER, M. C., M.B., Ch.B. Brist. .. .. .. | Ingledene, High Street, Staple Hill, Bristol. |
| *BARBOUR, R. F., M.A. Cantab., M.B., Ch.B. | 16 Mortimer Road, Clifton, Bristol. |
| *BARKER, G. H., M.D., B.Sc. Lond. .. .. .. | 124 Redland Road, Bristol 6. |
| *BATES, R. M. .. .. .. .. .. .. .. .. | Purdown House Stapleton, Bristol 5. |
| *BAXTER, V. T., M.B., Ch.B. .. .. .. .. | 167 Wells Road, Knowle, Bristol 4. |
| BAXTER, URSULA, M.B., Ch.B. Bristol .. .. | 167 Wells Road, Knowle, Bristol 4. |
| BEATSON, D. H., M.B., Ch.B. Brist. .. .. | 8 Richmond Park Road, Clifton, Bristol 8. |
| *BELL, R. J. IRVING .. .. .. .. .. .. .. | 5a Oakfield Road, Bristol. |
| *BERGIN, F. G. .. .. .. .. .. .. .. .. | 31 Oakfield Road, Clifton, Bristol 8. |
| *BERRY, R. J. A., Prof., M.D., C.M. Edin. .. | Director of Medical Services, Stoke Park Colony, Bristol 5. |
| *BIRRELL, J. A., M.D., B.S. Lond. .. .. .. | 1 Victoria Square, Bristol 8. |
| *BLACHFORD, J. V., *C.B.E.*, M.D. Durh. .. .. | Milverton House, Long Ashton, near Bristol. |
| BLACKETT, J. F., M.D., B.S. Lond. .. .. .. | Hamsterley, Bloomfield Park, Bath. |
| BLETCHLY, G. PLAYNE, M.B. Lond. .. .. | c/o Lloyds Bank, Nailsworth, Glos. |
| *BODMAN, A. P., M.B., Ch.B. .. .. .. .. | 11 Hurle Crescent, Clifton, Bristol 8. |
| *BODMAN, C. O., M.D. Durh. .. .. .. .. | 17 Upper Belgrave Road, Clifton, Bristol 8. |
| *BODMAN, F. H., M.D., Ch.B. Brist. .. .. .. | 2 Redland Court Road, Bristol 6. |
| BOLT, R. F. .. .. .. .. .. .. .. .. .. | 70 Great Russell Street, London, W.C.1. |
| *BOULTON, B. J., M.B., Ch.B. Brist. .. .. | Ham Green Hospital, Pill, near Bristol. |
| *BOWN, NAOMI J., M.B., Ch.B. Brist. .. .. | Demeter, Wraxall, Somerset. |
| *BRADBEER, E. G., M.B., Ch.B. Brist. .. .. | 13 Percival Road, Bristol 8. |
| BRASHER, C. W. J. .. .. .. .. .. .. .. | 62 Queen Anne St., Cavendish Square, London, W.1. |
| *BRINCKMAN, WINIFRED P., M.B., Ch.B. Brist. | 4 Oakland Road, Redland, Bristol 6. |
| *BRITTON, R. B., M.B., B.S. Lond. .. .. .. | 33a Whiteladies Road, Clifton, Bristol 8. |
| *BROCKLEHURST, R. J., M.A., D.M. Oxon. .. | The University, Bristol 8. |
| BROWN, J. E., M.B., Ch.B. Brist. .. .. .. | Coulthurst Villa, Brives Road, Maraval, Trinidad, B.W.I. |
| *BROWNE, R. C. .. .. .. .. .. .. .. .. | General Hospital, Bristol. |

| | |
|---|---|
| *BURGES, R. .. .. .. .. .. .. .. .. .. .. | Druid's Mead, Stoke Bishop, Bristol 9. |
| *BURGESS, N. F. C., M.D. Cantab. .. .. .. | Hereford House, Clifton, Bristol 8. |
| *BUSH, G. B., M.B., Ch.B. Brist. .. .. .. | Litfield House, Clifton, Bristol 8. |
| *CAIRNS, F. J... .. .. .. .. .. .. .. .. .. | Devon House, Whitehall Road, Bristol 5. |
| *CAPPER, W. M., M.B., B.S. Lond... .. .. | Bristol Royal Infirmary, Bristol. |
| *CAREY, R. S., *O.B.E.*, B.A. Cantab. .. .. | 502 Bath Road, Brislington, Bristol 4. |
| *CARLETON, H. H., M.A., M.D., B.Ch. Oxon. | 1 Lansdown Road, Clifton, Bristol 8. |
| *CASSIDY, A. D., M.B., B.Ch., B.A.O. .. .. | Bristol Children's Hospital, Bristol. |
| *CASSON, E., M.D., Ch.B. Brist. .. .. .. .. | Dorset House, Clifton Down, Bristol 8. |
| *CATES, H. J., M.D. Lond. .. .. .. .. .. | Northwoods House, Winterbourne, Bristol. |
| *CHAMBERS, E. R. .. .. .. .. .. .. .. .. .. | 9 Clifton Park, Clifton, Bristol 8. |
| *CHARLES, J. R., M.A., M.D. Cantab. .. .. | Litfield House, Clifton Down, Bristol 8. |
| *CHITTY, H., M.S. Lond. .. .. .. .. .. .. | 46 Pembroke Road, Clifton, Bristol 8. |
| *CHRISTOFFERSON, P. E., M.B., Ch.B. Brist. .. | 4 Victoria Square, Clifton, Bristol 8. |
| *CLAREMONT, L. E., M.D.S. Brist. .. .. .. | 5 Rodney Place, Clifton, Bristol 8. |
| *CLARK, DENNIS O. .. .. .. .. .. .. .. .. | 168 Milton Road, Bristol Road, Weston-super-Mare. |
| *CLARKE, R. C., *O.B.E.*, M.B., Ch.B. Brist. .. | 29 Victoria Square, Clifton, Bristol 8. |
| *CLUTTERBUCK, E. R., M.B., Ch.B. Brist. .. | 63 Walliscote Road, Weston-super-Mare. |
| *COATES, H. .. .. .. .. .. .. .. .. .. .. | Mental Hospital, Fishponds. |
| *COCHRANE, J. H. .. .. .. .. .. .. .. .. | 11 Bryant's Hill, St. George, Bristol 5. |
| *COLLINGWOOD, F. C., M.B., Ch.B. Brist. .. | 7 Hurle Crescent, Clifton, Bristol 8. |
| CONNELLAN, P. S... .. .. .. .. .. .. .. .. | Marmion, Shortheath Road, Farnham, Surrey. |
| *COOKE, ELIZABETH M., M.D. Lond. .. .. | Failand Lodge, Guthrie Road, Clifton. |
| *COOKE, R. V., Ch.M. Brist. .. .. .. .. .. | Failand Lodge, Guthrie Road, Clifton. |
| *COOKSON, R. G. .. .. .. .. .. .. .. .. .. | 5 Worcester Road, Clifton, Bristol 8. |
| *COOMBS, C. F., B.Ch. Cantab. .. .. .. .. | 14 Southfield Road, Westbury-on-Trym, Bristol. |
| COOMBS, N. C. .. .. .. .. .. .. .. .. .. | Romaldkirk, Barnard Castle, Co. Durham. |
| *COOPER, W. R. .. .. .. .. .. .. .. .. .. | 13 Westfield Park, Redland, Bristol 6. |
| *CORFIELD, C., M.D. Durh. .. .. .. .. .. | 5 Kensington Place, Clifton, Bristol 8. |
| *CORNER, BERYL, M.D., B.S. Lond. .. .. .. | 3 Elton House, Rodney Place, Bristol 8. |
| *COSSHAM, W. L., M.B., Ch.B. Brist. .. .. | 3 Claremont Road, Bishopston, Bristol 7. |
| *COURTNEY, R. M., M.B., Ch.B. Glasg. .. .. | Manor House, Southmead Road, Bristol. |
| *COXON, BEATRICE .. .. .. .. .. .. .. .. | 16 Richmond Hill, Clifton, Bristol 8. |
| *CRAIG, ANNE A., M.B., B.S. Lond. .. .. .. | 18 Vyvyan Terrace, Clifton, Bristol 8. |
| *CROOK, B. A., M.B., Ch.B. Brist. .. .. .. | The Laurels, Timsbury, near Bath. |
| *CROSSMAN, F. W., M.B. Durh. .. .. .. .. | White's Hill, Hambrook, near Bristol. |
| *DATTA, S. S., M.D. Brist. .. .. .. .. .. | Snowdon House, Fishponds, Bristol. |
| *DAVIES, E. D. D. .. .. .. .. .. .. .. .. | Standish House, Stonehouse, Glos |
| *DAWSON, W. R., *O.B.E.*, M.D. Dub. .. .. | 18 Brock Street, Bath. |
| *DELANEY, N., M.B., Ch.B. Manch. .. .. .. | The Meade, Bishopsworth, Somerset. |
| *DELICATI, J. L. .. .. .. .. .. .. .. .. | Royal Mineral Water Hospital, Bath. |
| *DICKSON, W. A., M.D. St. And. .. .. .. | Standish House, Stonehouse, Glos. |
| *DISNEY, M., M.D. Lond. .. .. .. .. .. .. | Bristol Royal Infirmary. |
| *DIX, C. .. .. .. .. .. .. .. .. .. .. .. | 14 Mortimer Road, Clifton, Bristol 8. |
| *DIXON, T. B. .. .. .. .. .. .. .. .. .. | 11 Pembroke Road, Clifton, Bristol 8. |
| *DODD, GRANTHAM, M.B., B.S., D.A., R.C.P., R.C.S. | 42 Woodstock Road, Bristol 6. |
| *DUERDEN, J. R., M.B., Ch.B. Brist. .. .. | 4 Cecil Road, Bristol 8. |
| *DUNLEVY, A. A., M.B., B.Ch. N.U.I. .. .. | 23 Pembroke Road, Clifton, Bristol. |
| *DUTTON, HILDA R. .. .. .. .. .. .. .. .. | 405 Stapleton Road, Bristol 5. |
| *DYER, W. P. .. .. .. .. .. .. .. .. .. | West View, Westbury-on-Trym, Bristol. |
| *EGLINTON, J. H. C. .. .. .. .. .. .. .. | Street, Somerset. |
| ELLIOTTS & AUSTRALIAN DRUG LTD. .. .. | 22 O'Connell Street, Sydney, N.S.W., Australia. |
| ELLIOTT, F. P., M.B., C.M. Aberd. .. .. .. | 113 Grove Road, Walthamstow, London, E. 17. |
| *ELLIOTT, W. H. A., M.B., B.S. Lond. .. .. | 116 Pembroke Road, Clifton, Bristol 8. |
| *ELLIS, W. T. .. .. .. .. .. .. .. .. .. | Tregenna House, Shirehampton. |
| *EVANS, G. M., M.B., Ch.B. Brist. .. .. .. | 117 Ashley Road, Bristol. |
| *EYRE-BROOK, A. L., M.B., M.S. Lond. .. .. | The Cottage, Portishead. |

| | |
|---|---|
| *FALLON, Col. J. .. .. .. .. .. .. .. .. .. | 35 Henleaze Gardens, Henleaze, Bristol. |
| *FAULL, J. L. .. .. .. .. .. .. .. .. .. | 61 Cotham Brow, Bristol 6. |
| *FAWN, G. F. .. .. .. .. .. .. .. .. .. .. | 6 Oakfield Road, Clifton, Bristol 8. |
| *FELLS, A., M.B., C.M. Edin. .. .. .. .. .. | 19 Alexandra Road, Clifton, Bristol 8. |
| *FELLS, G. R., M.B., Ch.B. Brist. .. .. .. .. | 19 Southmead Road, Bristol. |
| *FELLS, R. R., M.B., B.S. Lond. .. .. .. .. | 17 Mortimer Road, Clifton, Bristol 8. |
| *FENELEY, G. L., M.B., Ch.B. Brist. .. .. | Englewood, Falcondale Road, Westbury-on-Trym. |
| *FINZEL, H. F. M., M.D. Brist. .. .. .. .. .. | Nare House, Birchwood Road, St. Anne's Park, Bristol 4. |
| *FIRTH, J. L., M.D., M.S. Lond. .. .. .. .. | 8 Victoria Square, Clifton, Bristol 8. |
| *FISHER, A. C., M.D. Brist. .. .. .. .. .. | Roan Antelope Mine, N. Rhodesia. |
| *FISHER, J. A., M.D., B.Sc. Brist. .. .. .. .. | Canynge Hall, Bristol 8. |
| *FITZGIBBON, G. M., M.D. Lond. .. .. .. .. | 75 Pembroke Road, Clifton, Bristol 8. |
| *FITZGIBBON, Mrs. L. P., M.B., B.S. Lond. .. | 75 Pembroke Road, Clifton, Bristol 8. |
| *FLEMMING, A. L., M.B., Ch.B. Brist. .. .. | 48 Pembroke Road, Clifton, Bristol 8. |
| *FORRESTER-BROWN, MAUD, M.D., M.S. Lond. | 22 Combe Park, Bath. |
| *FORSTER, DAIREEN .. .. .. .. .. .. .. .. | Northam House, Henleaze Road, Bristol. |
| *FOSS, G. L., M.B., B.Ch. Cantab. .. .. .. .. | Cloud's Hill House, St. George, Bristol 5. |
| *FOX, F. E., B.A. Cantab. .. .. .. .. .. .. | Brislington House, Brislington, Bristol 4. |
| *FRASER, A. D., M.A., M.B., Ch.B. Glasg. .. | 4 Alexandra Road, Clifton, Bristol 8. |
| *FRASER, D., M.B., Ch.B. Edin. .. .. .. .. | Westgate Dursley, Glos. |
| *FREEMAN, RAYMOND .. .. .. .. .. .. .. .. | 6 Rutland Road, Wanstead, Essex. |
| *FULLER, H. L. .. .. .. .. .. .. .. .. .. .. | 9 Gay Street, Bath. |
| *FURNIVAL, Col. C. H. .. .. .. .. .. .. .. | "Yatton," Wellington Hill, Horfield, Bristol 7. |
| *GARDEN, R. R., M.A., M.B., B.Ch. Aberd. .. | 9 Harley Place, Clifton, Bristol 8. |
| GARLAND, E. B., M.B., C.M. Edin. .. .. .. .. | 114a Hampton Road, Redland, Bristol 6. |
| *GERRISH, NORMAN, M.B., Ch.B. Brist. .. .. | 2 Station Road, Keynsham, Som. |
| *GIBB, HELEN M., M.B., Ch.B. Glasgow .. .. | 10 Hill View, Henleaze, Bristol. |
| GIBBS, J. R. .. .. .. .. .. .. .. .. .. .. | 48 Codrington Road, Bishopston, Bristol 7. |
| *GLENNY, E. T., M.B., B.S. Lond. .. .. .. .. | Forester's Lodge, near Portishead, Som. |
| *GORDON, R. G., M.D., B.Sc. Edin. .. .. .. .. | 2 Catharine Place, Bath. |
| *GORHAM, A. P., M.B., Ch.B. Brist. .. .. .. .. | 53 Westbury Road, Westbury-on-Trym. |
| *GORNALL, W. A., M.D. Brist. .. .. .. .. .. | Ulvik, Nailsea, near Bristol. |
| *GREEN, S. B., M.B. Lond. .. .. .. .. .. .. | The Black Wicket, Westbury-on-Trym. |
| *GREENLAW, MADGE E., M.B., Ch.B. Brist. .. | 194 Redland Road, Bristol 6. |
| GRIFFITHS, CORNELIUS A. .. .. .. .. .. .. | 35 Newport Road, Cardiff. |
| *GROVES, E. W. HEY, M.D., M.S., D.Sc. Lond. .. | 25 Victoria Square, Clifton, Bristol 8. |
| *GUIRDHAM, A., M.A., B.M., B.Ch. Oxon. .. | Bailbrooke House, Bath. |
| *HALL, E. GEORGE, M.B. Lond. .. .. .. .. .. | 42 Apsley Road, Clifton, Bristol 8. |
| *HAM, C. I. M.B., Ch.B. Brist. .. .. .. .. .. | Frenchay Park Sanatorium, Frenchay, near Bristol. |
| *HARRIS, H. ELWIN, M.A., M.B. Cantab. .. | The Mount, Halse, Taunton. |
| *HARRIS, H. E., M.A., M.B., B.Ch. Cantab. .. | 13 Lansdown Place, Clifton, Bristol 8. |
| *HARTLEY, G., M.B., B.S. Lond. .. .. .. .. | 49 Queen's Court, Clifton, Bristol 8. |
| *HEALES, J. N. .. .. .. .. .. .. .. .. .. | Holmcroft, Street, Somerset. |
| *HECTOR, F., M.D. Lond. .. .. .. .. .. .. | 1 Victoria Square, Clifton, Bristol 8. |
| *HEMPHILL, R., M.B., B.Ch. Dub. .. .. .. .. | Molitor, Barrow Gurney, near Bristol. |
| *HENDERSON, JAMES, M.B., Ch.B. Aberd. .. | The City Sanatorium, Yardley Road, Birmingham. |
| *HERAPATH, C. E. K., *M.C.*, M.D. Lond. .. | Ormlie, Clifton, Bristol 8. |
| *HERON, GORDON, M.B., Ch.B. Brist. .. .. | 26 Henbury Road, Westbury-on-Trym, Bristol. |
| *HERON, DORIS, M.B., Ch.B. Brist. .. .. .. .. | 26 Henbury Road, Westbury-on-Trym, Bristol. |
| *HEWER, Professor T. F., M.D. Brist. .. .. | Canynge Hall, Bristol 8. |
| *HILL, WINIFRED, M.B., Ch.B. Brist. .. .. | 49 Tudor Gardens, West Acton W. 3. |
| *HOFFMAN, H. LOVELL, B.A., M.B., B.Ch. Cantab. | 26 The Circus, Bath. |
| *HOLLAND, W. A. L. .. .. .. .. .. .. .. .. | 10 Brunswick Square, Bristol. |
| *HOOKER, J. A., M.B., Ch.B. Brist. .. .. .. .. | 16 St. Edyth's Road, Sea Mills 9. |
| *HOSKEN, J. G. F... .. .. .. .. .. .. .. .. | Uley, Glos. |

HOSPITAL, BRISTOL GENERAL (*Secretary*, T. W. GREGG).

| | |
|---|---|
| *Houghton, Lydia M., M.B., B.S. Lond. | 74 Church Road, Redfield, Bristol 5. |
| *Howard, C. R. Grenville, M.B. | 8 Mortimer Road, Clifton, Bristol 8. |
| *Howell, H. | Kincraig, Beach Road, Weston-super-Mare. |
| *Hughes, F. W. Terrell, M.B., Ch.B. Brist. | Chew Magna, Somerset. |
| *Hughes, Marguerite G., M.B., Ch.B. Brist. | 12 Upper Belgrave Road, Clifton, Bristol 8. |
| *Hunter, F. S. | c/o Messrs. J. Wright & Sons Ltd., Publishers, Bristol 1. |
| *Hyatt, Annie W., M.B., B.S. Lond. | Merrymead, Shepton Mallet, Som. |
| *Iles, Anna, M.B., Ch.B. (Brist.) | 87 Pembroke Road, Clifton, Bristol 8. |
| *Iles, A. E., *O.B.E.*, M.B., B.S. Lond. | 87 Pembroke Road, Clifton, Bristol 8. |
| Infirmary, Bristol Royal (*Secretary*, E. C. Smith). | |
| Ingle, C. Durban | Somerton, Somerset. |
| *Jackman, W. A., M.B., Ch.B. Brist. | 15 Mortimer Road, Clifton, Bristol 8. |
| *James, April D., M.B., Ch.B. Brist. | |
| *James, J  M.D. Lond. | Litfield House, Clifton Down, Bristol 8. |
| *James, J. A  enr. | 43 Nevil Road, Bishopston, Bristol. |
| *Jenkins, F. G., M.B., Ch.B. Brist. | 51 Redcliff Hill, Bristol 1. |
| *Jenkins, R. D. | The Copper Beeches, Almondsbury, near Bristol. |
| *Jenkins, P. K., M.B., Ch.B. | 29 Downs Park West, Bristol 6. |
| *Johnson, R. G., M.B. Lond. | 119 Redland Road, Bristol 6. |
| *Joll, C. A., M.D., M.S. Lond. | 64 Harley Street, London, W.1. |
| *Jordan, L. R., M.B., Ch.B. Brist. | National Temperance Hospital, Hampstead Road, N.W.1. |
| *Joscelyne, P. C., M.B., Ch.B. Brist. | 17 Falcondale Road, Westbury-on-Trym. |
| *Kemm, N. F., M.B., B.S. Lond. | 200 Redland Road, Durdham Park, Bristol, 6. |
| *Kersley, G. D., M.D. | 6 The Circus, Bath. |
| King, E. F. | 2 Upper Wimpole Street, London, W.1. |
| *Kingston, S. H., M.B., Ch.B. Brist. | 3 Whiteladies Road, Clifton, Bristol 8. |
| *Kyle, H. G., M.A., M.D., B.Ch. Oxon. | 31 Westbury Road, Bristol. |
| *Lace, F | 5 Gay Street, Bath. |
| Lalonde, T. P., M.B., Ch.B. Brist. | Wykeham House, Romsey, Hants. |
| *Lees, E. Leonard, M.D., C.M. Edin. | 28 Downs Park West, Bristol 6. |
| *Legat, R. Eddowes, M.B., C.M. Edin. | 1 Cambridge Mansions. Woodland Road, Bristol 6. |
| *Lewis, F. J., M.B., Ch.B. Brist. | Canynge Hall, Bristol 8. |
| *Ling, T. M., M.A., B.M., B.Ch. | 50 Pembroke Road, Bristol 8. |
| *Linton, Marion S., M.B. Lond. | 1 Brecon Road, Henleaze, Bristol. |
| *Lister, A. E. J., M.B., B.S. Lond. | 86 Pembroke Road, Clifton, Bristol 8. |
| *Lister, L. Margaret | 12 All Saints Road, Clifton, Bristol 8. |
| *Logan, H. B. | Elmwood, Aberdeen Road, Cotham, Bristol 6. |
| *Loxton, S. D., M.B., Ch.B. | 17 Oak eld Grove, Clifton, Bristol 8. |
| *Lucas, J. E. | 48 Coronation Road, Bristol 3. |
| *Lucas, J. J. S., M.D. Lond. | 186 Wells Road, Totterdown, Bristol 4. |
| *Lucas, J. V. | 186 Wells Road, Knowle, Bristol 4. |
| *Lucas R. P., M.B., Ch.B. Brist. | 15 Edgecumbe Road, Redland Bristol 6. |
| *McCrea, Phillip, M.D., B.Ch. Dub. | 56 Kellaway Avenue, Bristol 6. |
| *McKenzie, W. G., *M.C.* | 10 Woodland Road, Surbiton. |
| *MacLachlan, A. M., M.B., Ch.B. Glasg. | 65 Redcliff Hill, Bristol 1. |
| *McLannahan, J. G. | The Mount, Stonehouse. |
| *MacLaren, Catherine J., M.B., Ch.B. (Edin.) | Hereford House, Clifton Down. |
| Maclean, Sir Ewen J., M.D., C.M. Edin. | 12 Park Place, Cardiff. |
| *MacLeod, Donald, M.B., C.M. Edin. | 84 Redland Road, Redland, Bristol 6. |
| *Macleod, G., M.A. Glas., M.D., M.B. | Wargrave, Clevedon, Somerset. |
| *Marshall, A. H., M.B., Ch.B. Brist. | Odelos, Canford Lane, Bristol. |
| Marshall, A. L., M.B., B.A. Cantab. | Laxfield, Woodbridge, Suffolk. |
| *Martin, J. E., M.B., Ch.B. | 32 Whiteladies Road, Clifton. |
| *Martin, J. J. B., M.A., M.D. Edin. | Bristol Mental Hospital. |
| *Martin, P. S., *M.C.* | Victoria Quadrant, Weston-super-Mare. |
| *Matheson, N. R., M.B., Ch.B. | Southmead Hospital, Bristol. |
| *Mayes, F. J. A. | 23 Pembroke Road, Clifton, Bristol 8. |
| *Meadows, G. | W. D. & H. O. Wills, No. 1 Factory, East Street, Bedminster, Bristol 3. |

| | |
|---|---|
| MIALL, C. L'O | Elm Hayes, Paulton, near Bristol. |
| *MILLING, T., M.B., Ch.B. Belf. | 1 Vicarage Road, Southville, Bristol 3. |
| *MOORE, C. A., M.B., M.S. Lond. | South Lodge, Kingsweston Road, Henbury, Bristol. |
| *MOORE, L. A., M.B., Ch.B. Brist. | Hillsborough, Cotham Park, Bristol 6. |
| *MOORE, R. H., M.B., Ch.B. Brist. | White Cross Court, Wells Road, Bristol 4. |
| *MORGANS, C. C., M.B., Ch.B. Cantab. | 65 Lower Redland Road, Bristol 6. |
| *MORRIS, L. N., M.B., Ch.B. Brist. | 13 Belgrave Road, Clifton, Bristol 8. |
| *MUNDY, G. S., M.B., B.Ch. Brist. | Homewood, Coombe Dingle, Bristol. |
| *MYLES, G. T. | 7 Apsley Road, Clifton, Bristol 8. |
| *MYLES, Q. St. L., M.A., B.M., B.Ch. Oxon. | 7 Apsley Road, Clifton, Bristol 8. |
| *NEWLANDS, H. J. | Moray House, Fishponds, Bristol. |
| *NICHOLSON-LAILEY, J. R. | Elmfield House, South Road, Taunton. |
| *NIXON, DOREEN, M.B., B.S. Lond. | 7 Lansdown Place, Clifton, Bristol 8. |
| *NIXON, J. A., *C.M.G.*, M.D. Cantab. | 7 Lansdown Place, Clifton, Bristol 8. |
| *NOBLE, J. I., M.B., Ch.B. Liverp. | 102 Pembroke Road, Clifton, Bristol 8. |
| *NORMAN, R. M., M.D. Brist. | 25 Victoria Square, Clifton, Bristol 8. |
| *NOTT, WINIFRED, M.B., Ch.B. Brist. | Fassiefern, 8 Rylestone Grove, Stoke Bishop, Bristol 9. |
| *O'DONOHOE, MONICA A., M.B., Ch.B. | 19 Westfield Park, Redland, Bristol 6. |
| *ORMEROD, G. L. M.A., M.B., B.Ch. Cantab | 2 Henleaze Road, Westbury-on-Trym, Bristol. |
| *ORR-EWING, H. J., *M.C.*, M.D. Lond. | 1 Beaufort Buildings, Clifton, Bristol 8. |
| *PALIN, A. G., B.M., B.Ch. Oxon. | 23 Pembroke Road, Bristol 8. |
| *PARKES, J. A., M.B., Ch.B. Liverp. | 8 South Road, Redland, Bristol 6. |
| PARKINSON, Lt.-Col. G. S., *D.S.O.*, R.A.M.C. | London School of Hygiene and Tropical Medicine, Keppel Street, London, W.C. 1. |
| *PARRY, R. H., M.B., B.S. Lond. | Public Health Offices, 40 Prince Street, Bristol 1. |
| PARSONS, Sir J. H., M.B., B.S., B.Sc. Lond. | 54 Queen Anne Street, London, W. |
| *PAUL, R. G. | 23 Pembroke Road, Bristol 8. |
| *PAULI, C. H. | Turville, Pilning. |
| *PERRY, B., M.D., Ch.B. Brist. | 4 Richmond Park Road, Clifton, Bristol 8. |
| *PHILIPP, E., M.B., Ch.B. | 521 Filton Avenue, Bristol 7. |
| *PHILLIPS, L. B., M.B., Ch.B. Brist. | Holdenhurst, Filton, Bristol. |
| *PHILLIPS, P., M.B., Ch.B. Brist. | Southmead Hospital, Bristol. |
| *PINEO, E. D. | Richmond House, Langford, Som. |
| *PIRRIE, A. L., M.B., Ch.B. Glasg. | 528 Kensington Hill, Bristol. |
| *POCOCK, J. A., M.A., M.B., B.Ch. Cantab. | General Hospital, Bristol. |
| *POLLARD, J., M.B., B.Ch., B.A.O., N.U.I | 88 Coronation Road, Bristol 3. |
| PORTER, C. P. | 28 Mill Street, Kidderminster. |
| *POTTER, MABEL F., M.B., Ch.B. Brist. | 79 Pembroke Road, Clifton, Bristol 8. |
| *POWELL, H. F., M.D. Brux. | Ellenborough Park, Weston-super-Mare. |
| *PRICE, C. H. G., M.B., Ch.B. Brist. | 6 Lansdown Place, Bristol 8. |
| *PRICE, N. L., M.B., Ch.B. Brist. | 12 The Paragon, Clifton, Bristol 8. |
| *PRIDIE, K. H., M.B., B.S. Lond. | 58 St. John's Road, Clifton, Bristol 8. |
| *PROWSE, D. C., M.B., Ch.B. Brist. | Wigmore House, Thornbury, near Bristol. |
| *PYKE, H. D., M.B., Ch.B. Brist. | 88 Redland Road, Bristol 6. |
| *RANKIN, P. C., M.B., Ch.B. Glasg. | Lawrence Hill House, Bristol. |
| *RAYNER, D. C., Ch.M. Brist. | 9 Lansdown Place, Clifton, Bristol 8. |
| *REDMAN, ETHEL, M.B., Ch.B. | 55 Bridgwater Road, Bedminster Down, Bristol 3. |
| *REES, W. G., MB., Lond. | Northam House, Henleaze Road, Bristol. |
| *ROBERTS, J. A. F., M.A. Cantab., M.B., Ch.B., D.Sc. Edin. | Stoke Park Colony, Bristol. |
| *ROBERTSON, D., M.B., Ch.B. Edin. | 2 Knowle Road, Knowle, Bristol 4. |
| *ROBERTSON, L. G., M.B., Ch.B. | 162 Redland Road, Bristol 6. |
| *ROGERS, H., M.D. Brist. | 91 Old Park Ridings, Grange Park, N.21. |
| *ROLFE, R., B.A., M.B., B.C. Cantab. | Park House, St. Andrew's Road, Avonmouth. |
| *ROWLANDS, R. D. | Elmfield House, South Road, Taunton, Som. |
| *ROWLEY, A. T. | 13 Walliscote Road, Weston-super-Mare. |
| *RUDOLF, G. de M. | 5 Pembroke Road, Clifton, Bristol 8. |
| *RYAN, P. B., M.B., Ch.B. | Southmead Hospital, Bristol. |

| | |
|---|---|
| *SAMPSON, O. E. L. | 77 Stackpool Road, Bristol 3. |
| *SCARFF, G., M.B., Ch.B. Edin. | 83 Pembroke Road, Clifton, Bristol 8. |
| *SHEPHERD, H. L., M.B., Ch.M. Brist. | 79 Pembroke Road, Clifton, Bristol 8. |
| *SHORT, A. R., B.Sc., M.D., B.S. Lond. | 69 Pembroke Road, Clifton, Bristol 8. |
| *SMITH, T. W., M.D. Lond. | 26 The Circus, Bath. |
| *SMYTHE, H. J. D., *M.C.*, M.D., M.S. Lond. | 15 Eaton Crescent, Clifton, Bristol 8. |
| *SNAWDON, J. W. E., M.B., Ch.B., B.D.S. | 170 Redland Road, Bristol 6. |
| *SOLTAU, H. K. V., M.D. Lond. | 19 The Avenue, Clifton, Bristol 8. |
| *SOMERS, MARY A. E. | 2 Ashcombe Road, Weston-super-Mare. |
| *SPOOR, A., M.B., B.Ch. Cantab. | The Hydro, Bristol 1. |
| *STANILAND, M. F. | 255 Stapleton Road, Bristol 5. |
| *STATHAM, R. S. S., *O.B.E.* M.D., M.Ch. Brist. | Cheddar, Som. |
| *STEVEN, G. D., M.B., Ch.B. Edin., D.P.H. | 6 Upper Church Street, Bath. |
| *STONE, DORIS M., M.D., B.S. Lond. | British Postgraduate School, Ducane Road Shepherds Bush, W.11. |
| *STROVER, H. W. M., *O.B.E.*, M.B., Ch.B. Aberd. | 90 Redland Road, Bristol 6. |
| *STRUTHERS, A. J., M.B., Ch.B. Glasg. | 100 Church Road, Redfield, Bristol 5. |
| *STRUTHERS, GEO., M.B., Ch.B. (Glasgow) | Southmead Hospital, Bristol. |
| *SUTTON, G. E. F., M.D. Lond. | 1 Pembroke Road, Clifton, Bristol 8. |
| *SYMES, J. O., M.D. Lond. | 6 Pembroke Vale, Clifton, Bristol 8. |
| *TASKER, D. G. C., M.B., M.S. Lond. | 9 College Fields, Clifton, Bristol 8. |
| *TAYLOR, A. L., M.D. Lond. | The General Hospital, Bristol 1. |
| TAYLOR, A. L. | The Royal Infirmary, Bristol 2. |
| *TEMPLE, G. H., M.B., C.M. Edin. | Ailanthus, Weston-super-Mare. |
| THIN, J. | 54 and 55 South Bridge, Edinburgh. |
| *THOMAS J. D., M.B. Cantab. | Dan-y-Graig, Cheyne Road, Stoke Bishop, Bristol 9. |
| *TODD, A. T., *O.B.E.*, M.D. Edin. | Royal Park House, Clifton, Bristol 8. |
| TRIPP, DOROTHY M. H. | Mission Hospital, Karim Nagar, Nizam's Dominions, South India. |
| *TRIST, J. R. R., *M.C.* | 120 Henleaze Road, Henleaze. |
| *TRYON, VICTORIA S., M.B., Ch.B. Brist. | 3 Harley Place, Clifton, Bristol 8. |
| *WALKER, CYRIL H., B.A., M.B. Cantab. | Harewood, Clapton-in-Gordano, Som. |
| *WALTERS, C. F. | Downover, Walton Down, near Clevedon. |
| *WANSBROUGH, T. B., M.B., Ch.B. Brist. | 8 Mortimer Road, Clifton, Bristol 8. |
| WAKE, S. C., M.B., Ch.B. Brist. | "Stanhope," Bideford, N. Devon. |
| *WARD, H. W. | Chipping Manor, Wotton-under-Edge. |
| *WARREN, R., M.A., M.D. Oxon. | Heathgates, Weston-super-Mare. |
| *WATERHOUSE, R,, M.D. Lond. | 3 The Circus, Bath. |
| *WATSON-WILLIAMS, E., *M.C.*, B.A., M.B., Ch.M. Cantab. | 65 Pembroke Road, Clifton, Bristol 8. |
| WATTS, J. B. V. | 6 Queen Street, Cambridge, C.P., South Africa. |
| WEDDELL, C. W. | Boydville, 46 Miller Street, West Melbourne, Australia. |
| *WIGODER, SYLVIA, M.A., M.D., Ph.D. | General Hospital, Bristol 1. |
| *WILBOND, R. G. | 76 Chesterfield Road, St. Andrew's Pk., Bristol. |
| *WILLIAMS, I., M.B., Ch.B. Brist. | "Ardrem," Hill View, Henleaze, Bristol. |
| *WILLIAMS, S. R., M.B. Lond. | Buckfastleigh, Devon. |
| *WILLWAY, F. W., M.D., M.S. Lond. | Bristol Royal Infirmary. |
| *WINTER, J. R., B.A., M.B., B.Ch. Cantab. | Kingsleigh, Cirencester, Glos. |
| *WOOD, D. | 105 Pembroke Road, Clifton, Bristol 8. |
| *WOOD, URSULA, M.B., Ch.B. | Henley Lodge, Yatton, Som. |
| *WOOD, W. V., *M.C.*, B.A. Cantab. | Henley Lodge, Yatton, Som. |
| *WOODMAN, DOROTHY, M.D. Lond. | Kynance, Downs Cote Park, Westbury-on-Trym, Bristol. |
| *WOOLLEY, A. W., M.B., Ch.B. Brist. | Melbourne House, Wells, Som. |
| *WOOLLEY, W., M.B., Ch.B. Brist. | 239 Cranbrook Road, Redland. |
| *WORTHINGTON, Lieut.-Col. F., M.B., Ch.B. Cantab. | Greytrees, Briercliffe Road, Coombe Dingle Bristol 9. |
| *WRIGHT, A. J., M.B., B.S. Lond. | 2 Clifton Park, Clifton, Bristol 8. |
| *WRIGHT, WINIFRED M., M.B., B.S. Lond. | Merrymead, Shepton Mallet, Som. |
| *ZEALAND, L., M.D. Man. | Brecon Lodge, Westbury-on-Trym, Bristol. |

